# Origin, Genetic Diversity, and Evolutionary Dynamics of Novel Porcine Circovirus 3

**DOI:** 10.1002/advs.201800275

**Published:** 2018-07-04

**Authors:** Gairu Li, Wanting He, Henan Zhu, Yuhai Bi, Ruyi Wang, Gang Xing, Cheng Zhang, Jiyong Zhou, Kwok‐Yung Yuen, George F. Gao, Shuo Su

**Affiliations:** ^1^ MOE International Joint Collaborative Research Laboratory for Animal Health & Food Safety Jiangsu Engineering Laboratory of Animal Immunology Institute of Immunology College of Veterinary Medicine Nanjing Agricultural University Tongwei Road, Xuanwu District Nanjing 210095 China; ^2^ MRC‐University of Glasgow Centre for Virus Research 464 Bearsden Road Glasgow G61 1QH UK; ^3^ CAS Key Laboratory of Pathogenic Microbiology and Immunology Institute of Microbiology Chinese Academy of Sciences NO.1 Beichen West Road, Chaoyang District Beijing 100101 China; ^4^ Key Laboratory of Animal Virology of Ministry of Agriculture Zhejiang University 866 Yuhangtang Rd Hangzhou 310058 China; ^5^ Department of Microbiology Queen Mary Hospital Hong Kong 999077 China; ^6^ National Institute for Viral Disease Control and Prevention Chinese Center for Disease Control and Prevention (China CDC) Beijing 102206 China

**Keywords:** bat circovirus, evolution, genotypes, phylodynamic, phylogenetic analysis, porcine circovirus 3 (PCV3), swine

## Abstract

Porcine circovirus 3 (PCV3) is a novel virus associated with acute PDNS (porcine dermatitis and nephropathy syndrome)‐like clinical signs identified by metagenomic sequencing from swine. Its high occurrence may pose a potential threat to the swine industry worldwide. The processes resulting in the emergence and spread of PCV3 remain poorly understood. Herein, the possible origin, genotypes, and evolutionary dynamics of PCV3 based on available genomic sequences are determined. The closest ancestor of PCV3 is found to be within the clade 1 bat CVs. Using different phylogenetic methods, two major genotypes are identified, PCV3a and PCV3b. It is found that the effective population size of PCV3 increased rapidly during late 2013 to early 2014 and this is associated with the diversification of PCV3a and PCV3b. A relatively high effective reproductive number (Re) value and higher evolutionary rate were found compared to other single‐stranded DNA viruses, and positive selection on codons 122 and 320 (24 of ORF2) is identified. It is hypothesized that this, together with the prediction of a potential change of an antigenic epitope at position 320, might have allowed PCV3 to escape from the host immune response. Overall, this study has important implications for understanding the ongoing PCV3 cases worldwide and will guide future efforts to develop effective preventive and control measures.

## Graphical abstract

**Porcine circovirus 3 (PCV3) is a focal point in the veterinary and swine industries** due to its worldwide prevalence and potential threat. There is insufficient knowledge of the epidemiology and evolution of PCV3 at present. PCV3 is linked to the contemporary bat‐origin clade 1, and an accurate evolution and genotypic characterization of PCV3 is provided. The highest substitution rate in single‐stranded DNA viruses and relatively high effective reproduction number indicate the possibility of a continuous outbreak in the future.

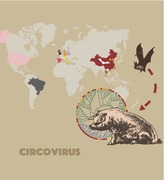

## 1 Introduction

Continuous epidemiological surveillance of emerging viruses is crucial to control widespread.^1^ According to the International Committee on Taxonomy of Viruses (ICTV), the family *Circoviridae* comprises two genera: *Cyclovirus* and *Circovirus*. Viruses from the genus *Cyclovirus* have been found in humans and chimpanzees.^2^ Circoviruses have a wide host range, including human,^2^, ^3^ birds,^4^, ^5^ pigs,^6^, ^7^ dogs,^8^ and cattle.^9^, ^10^ Before to 2015, porcine circovirus 1 (PCV1) and PCV2 were considered the only types of porcine circoviruses. PCV1 is nonpathogenic to pigs and was identified in the porcine kidney cell line PK‐15.^6^, ^11^ PCV2 is associated with significant clinical signs such as postweaning multisystemic wasting syndrome (PMWS), porcine circovirus‐associated diseases (PCVAD), and immunosuppression.^2^, ^6^, ^10^ In the late 1990s, the first PCV2 outbreak was identified^12^, ^13^ and soon PCV2 became endemic causing severe economic losses in the swine industry. Its high evolutionary rate, the highest recorded for a single‐stranded DNA virus until now, could allow an evolutionary dynamics similar to that of single‐stranded RNA viruses.^13^ The genotype identification of PCV2 was first proposed by the European project in 2008 based on *p*‐distance of neighbor joining (NJ) trees.^14^ However, it was questioned by Franzo et al. and others^12^, ^15^, ^16^ when more sequences became available. Until now, there are three major confirmed genotypes including: PCV2a, PCV2b, and PCV2d, and two genotypes with low prevalence, PCV2c and PCV2e.^16^, ^17^, ^18^, ^19^ Genotype shifts may be associated with differences in pathogenicity and vaccine immunity.^12^ New standard methods to define PCV2 genotypes are necessary to adapt vaccines to the rapid emergence of new genotypes.^20^

Although PCV2 is the cause of big economic losses to the pig industry, there is uncertainty surrounding its origin and evolution. Recently, a novel circovirus was identified by next generation sequence (NGS) analysis of aborted fetuses of sows and named PCV3.^21^, ^22^ The newly discovered virus was associated with PDNS.^23^ PCV3 infection might contribute to PDNS and reproductive failure, cardiac, and multisystemic inflammation.^10^, ^21^, ^24^ It has also been shown that PCV3 found in aborted fetuses is the result of vertical transmission.^21^ Like PCV2, there are two major open reading frames (ORFs) in the genome of PCV3: ORF1 encodes proteins involved in viral replication (Rep) and ORF2 encodes a major structural protein, the capsid protein (Cap).^25^ PCV3 sequences were detected widely in the USA,^21^ China,^26^, ^27^, ^28^ Brazil,^29^ Thailand,^30^ Korea,^23^ and many European countries,^22^, ^31^, ^32^, ^33^, ^34^ including Poland, Italy, Spain, Denmark, Germany, the UK among others. The PCV3 that was first identified in the USA was more closely related to canine circovirus although with weak bootstrap support.^21^ Subsequently, with PCV3 detected in China and other countries, a study reported that it was closely related to some bat circoviruses.^24^ Additionally, it was considered that canine CVs and some bat CVs may share a common ancestor within the cluster containing PCV1 and PCV2.^35^ Previous studies on the origin of PCV3 are difficult to interpret and compare since different phylogenetic methods and reference sequences were applied.^21^, ^24^, ^26^

Although phylogenetic analysis is a powerful tool and is now widely used for investigating the evolution of PCV3, there are a large number of different inference methods and a lack of uniformity in the use of these methods by different studies.^22^, ^24^, ^25^, ^26^, ^27^, ^29^, ^31^, ^33^ Considering the controversy of the origin of PCV3 (bat or canine),^21^, ^24^, ^26^ we analyzed the selected available ORF1 circovirus sequences using maximum‐likelihood (ML) and Markov chain Monte Carlo (MCMC) methods to trace more accurately the origin of PCV3. Additionally, all available complete PCV3 sequences deposited in GenBank until November 2017 were analyzed by NJ, ML, and MCMC methods to study the molecular genetic relationship. Our results provide a global view of the origin, genetic divergence, and evolutionary dynamics of PCV3 and indicate positive selection and high evolutionary rate, supporting the ongoing genotype shift and outbreaks.

## 2 Results

### 2.1 Temporal Evidence for the Origin of the Emergent PCV3 Viruses Worldwide

**Figure 1:**
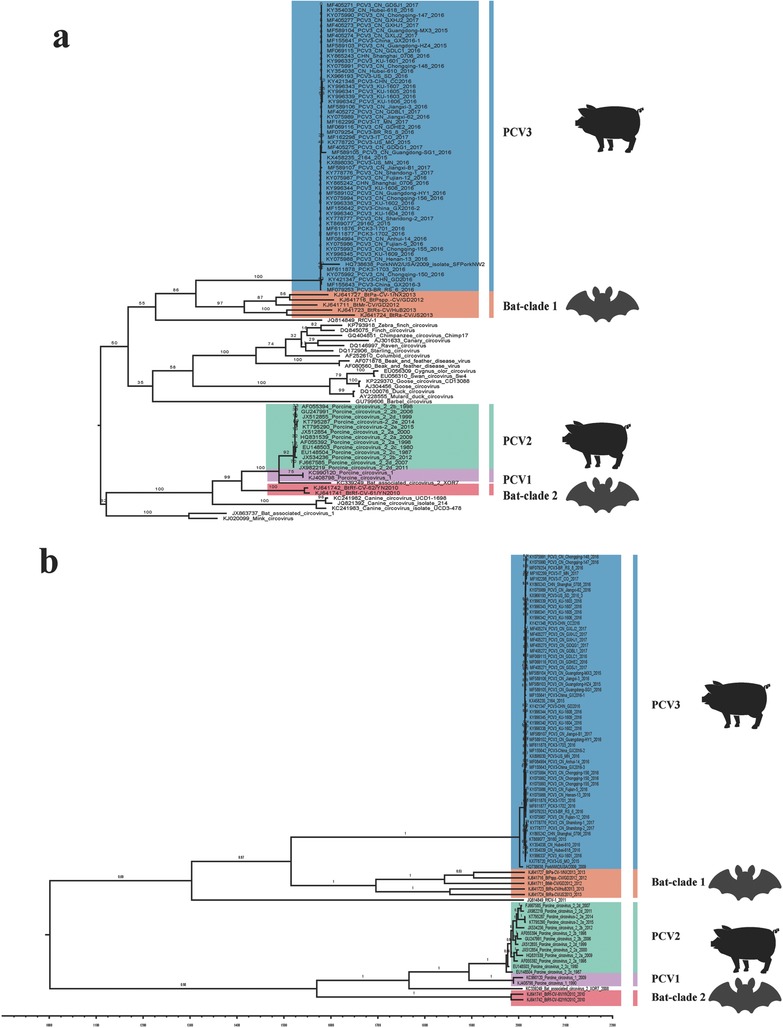
The origin of PCV3 was deduced using the conserved coding region of ORF1. a) ML tree reconstructed using RAxML of 101 ORF1 genes including different species of circoviruses. b) MCC tree reconstructed using BEAST (v1.8.4) with 81 of the 101 strains. The posterior displayed along each branch. Different clades represented by different colors as displayed in the figures.

RDP4 was used to detect recombination. We detected no recombination. However, the KY418606 sequence was removed due to low quality. To trace the origin of PCV3, a ML tree (**Figure** **1**a) was reconstructed using ORF1 gene sequences. We found that all the PCV3 strains were closely related to the clade 1 bat CVs (which were isolated in China from 2011 to 2013) with a high confidence (bootstrap = 86). The bat virus *Rhinolophus ferrumequinum* circovirus 1 from China (JQ814849) acted as an outgroup of the clade 1 bat CVs and the PCV3 strains, indicating a potential bat CV origin. Additionally, we also found that the PCV2 and PCV1 strains were closely related to the clade 2 bat CVs in the study of Wu et al.^35^ We also discovered that canine CVs are closely related to clade 2 bat CVs and PCV1 and PCV2 strains. However, it was previously reported that canine CVs were closely related to PCV3 strains when comparing complete genomes, although with low bootstrap values.^21^ This supports the idea that the conserved Rep protein should be used when inferring the origin of porcine circoviruses in the future. Furthermore, the maximum clade credibility (MCC) tree reconstructed with 81 of the 101 ORF1 sequences (Figure 1b) showed similar topology with the ML tree, and evidenced a close relationship with clade 1 bat CVs with uncertain divergence time due to the insufficient numbers of sequences currently available (Table S1, Supporting Information).

### 2.2 PCV3 Genotyping According to Phylogenetic Analysis

**Figure 2:**
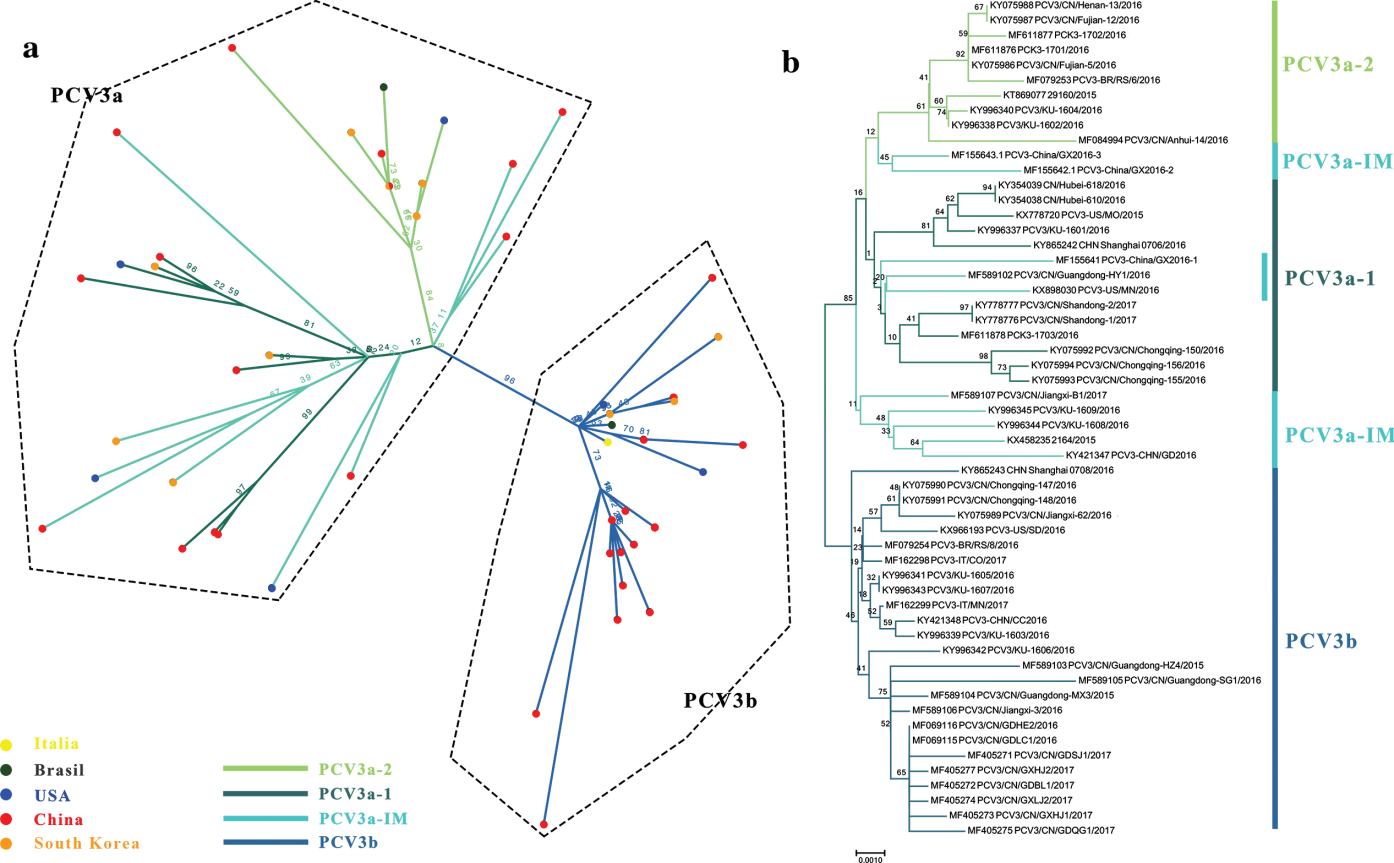
Phylogenetic analysis of the available 56 complete coding sequences. a) ML reconstructed tree using RAxML. b) NJ tree reconstructed using MEGA 7.0. The values along the branches represent bootstrap values. The different genotypes are represented by different colors as indicated in the figures.

**Figure 3:**
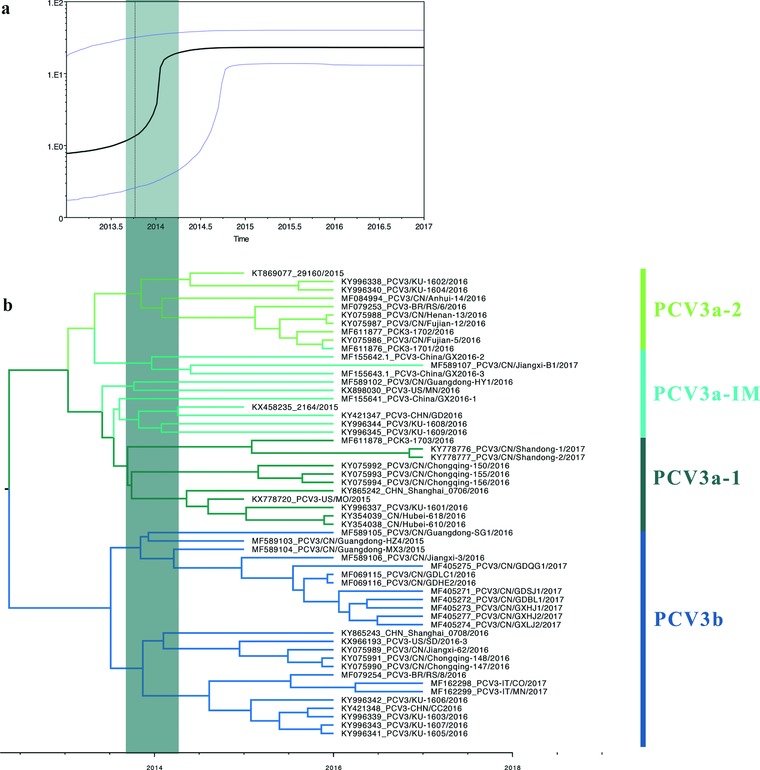
Bayesian skyline plot showing changes in genetic divergence of PCV3 complete coding sequences. a) A measure of genetic diversity is given on the *y*‐axis with the 95% HPD shown in blue. b) MCC tree scaled to time using the GTR+I+G substitution model and an uncorrected relaxed clock (lognormal) of PCV3 complete coding sequences. The green rectangles in both panels indicate the potential divergence period of PCV3 complete coding sequences.

We used ML, MCC, and NJ methods to reconstruct the phylogenies of PCV3 complete coding sequences (ORF1+ORF2). Two independent clades were observed in the three different trees which displayed similar structures (**Figure** **2**a,b and **Figure** **3**b). Interestingly, detailed analysis of the distribution of the sequences in the distinct clades revealed that the sequence structures were similar using the different algorithms, except for several branches in one clade (PCV3‐China/GX2016‐3, PCV3‐China/GX2016‐2, PCV3/CN/Guangdong‐HY1, PCV3‐China/GX2016‐1, PCV3‐US/MN, PCV3/CN/Jiangxi‐B1, PCV3/KU‐1609, PCV3/KU‐1608, 2164, PCV3‐CHN/GD). Considering the stable structures of the complete coding sequences, we named them clades PCV3a and PCV3b, respectively. The PCV3a clade included the strains first identified in the USA in 2015 (PCV3‐US/MO/2015).^21^ Moreover, the PCV3a clade could be separated into two individual subclades with stable structures, termed PCV3a‐1 and PCV3a‐2. We observed that the distribution of several strains was random in the PCV3a clade and we referred to these strains as intermediate strains (IM).

However, the NJ and ML tree constructed using ORF2 did not display clear clusters, especially in NJ tree. For example, in the NJ tree constructed with 109 ORF2 sequences, some of the strains in PCV3a‐IM clustered with PCV3b (Figure S1, Supporting Information). The phylogenies did not remain consistent when the number of reference sequences of the ORF2 gene increased. Therefore, we suggest using complete coding sequences for PCV3 genotyping.

### 2.3 Evolution and Epidemiological Dynamics Analysis of PCV3

**Figure 4:**
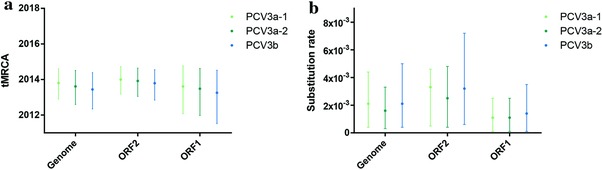
The tMRCAs and substitution rates were calculated in BEAST (v1.8.4) for ORF1, ORF2, and the complete coding sequences of PCV3a‐1, PCV3a‐2, PCV3‐IM, and PCV3b. The different genotypes are represented by different colors.

The time of most recent ancestor (tMRCA) and nucleotide substitution rate of the complete coding sequences and individual genes were estimated using BEAST (v1.8.4). Both the mean value and 95% HPD (high posterior destiny) were included (**Figure** **4**). The tMRCA of PCV3a was 2013.04 (95% HPD: 2011.69–2013.99). In detailed, the estimations of the tMRCA of PCV3a‐1, PCV3a‐2, and PCV3b were 2013.80, 2013.61, 2013.44, respectively, which suggests that the PCV3a clade experienced a rapid expansion in 2013. Additionally, the tMRCA of ORF2 and ORF1 was also estimated and showed a similar pattern with complete coding sequences (Table S2, Supporting Information). Overall, the mean tMRCA of PCV3 of the last three years was in late 2013 based on individual gene or complete coding sequence analysis.

Additionally, the mean substitution rate of the complete coding sequences of all the strains was 1.69 × 10^−3^ substitutions/site/year, which was higher than previously reported for PCV1 (1.15 × 10^−5^),^36^ but closer to PCV2 (1.2 × 10^−3^) (Table S2, Supporting Information).^13^ Furthermore, to get more detailed information on the phylogenetic evolution of PCV3, the substitution rates were estimated according to specific clades and the individual genes. As shown in Figure 4, the substitution rates of different clades in terms of the complete coding sequences were 2.1 × 10^−3^ in the PCV3a‐1 and PCV3b clades and 1.6 × 10^−3^ in the PCV3a‐2 clade. Additionally, we observed that the ORF2 gene experienced similar evolution compared to the ORF1 gene (Table S2, Supporting Information).

Based on the skyline plot and MCC tree, we found that PCV3 might have diversified from the second half of 2013 to the first half of 2014 (Figure 3a). In addition, the various branches of PCV3 (PCV3a‐1, PCV3a‐2, and PCV3b) also formed around this time (Figure 3b).

Moreover, we investigated the epidemiological dynamics of emerging PCV3 worldwide. We found considerable temporal variation in the estimates of the effective reproductive number (Re) using sequences from 2015–2017. Despite distinct phylogenetic histories, PCV3a and PCV3b exhibited similar Re values of 3.08 (PCV3a, 95% HPD: 0.82–7.96) and 1.82 (PCV3b, 95% HPD: 0.63–4.86) (Figure S2, Supporting Information) based on epidemiological modeling in 2016. This suggests the potential to become endemic in pigs, at least if compared to human influenza viruses.^37^ Considering the limited number of sequences for 2015 and 2017, the Re value of 2016 was considered valid.

The association of geographical distribution of PCV3 indicated that the *p* value of both association index (AI) and parsimony score (PS) were less than 0.05. The *p* value of monophyletic clade (MC) observed in China was less than 0.05 (**Table** **1**), indicating that the geographic relevance of PCV3 in China is significant (a significant association was also observed in Italy but a low number of sequences was analyzed). However, the *p* values of the USA, South Korea, and Brazil were greater than 0.05. The association of the geographical distribution of PCV3 is displayed in the Figure S3 (Supporting Information) map. In general, there was a correlation between PCV3 and geography, although the correlation was weak based on the relatively small number of available sequences.

**Table 1:** BaTS analysis: correlation analysis among geography and PCV3 strains

Statistic	Observed mean (95% *HPD)	Null mean (95% *HPD)	**p‐value
AI	1.87(1.43,2.32)	3.45(2.88,4.05)	0
PS	16.66(16.00,18.00)	19.74(18.34,20.8)	0
MC (USA)	1(1.00,1.00)	1.1(1.00,1.54)	1
MC (China)	11.95(12.00,12.00)	4.34(3.03,8.20)	0.01
MC (Korea)	2.28(2.00,3.00)	1.65(1.09,2.26)	0.23
MC (Brazil)	1(1.00,1.00)	1(1.00,1.01)	1
MC (Italy)	1.63(1.00,2.00)	1(1.00,1.00)	0.01

### 2.4 Amino Acid Analysis of the Related Clades and Predicted Immune Epitopes

**Figure 5:**
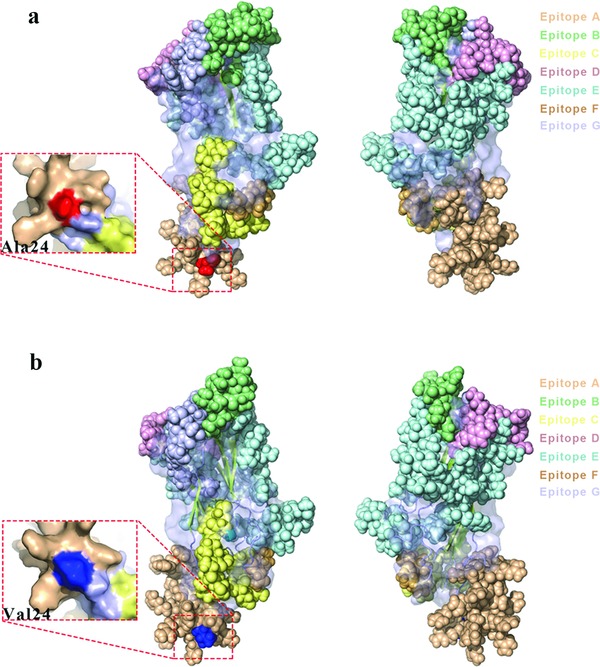
Prediction of immune‐epitopes of the reference strains of a) PCV3a and b) PCV3b. The different predicted epitopes in the ORF2 protein of both PCV3a and PCV3b are indicated by different colors: epitope A (wheat), epitope B (pale green), epitope C (pale yellow), epitope D (light pink), epitope E (pale cyan), epitope F (light orange), and epitope G (blue white). Amino acid site 24 in ORF2 (site 320 on the complete coding sequences) of PCV3a and PCV3b is indicated.

A total of 56 PCV3 complete coding sequences were used for amino acid analysis of the related clades (Table S3, Supporting Information). Amino acid sites 122, 320, and 323 were crucial to distinguish PCV3a and PCV3b. Compared to PCV3a, these amino acid sites were S122A, A320V, R323K, respectively, in PCV3b while R323K in PCV3a‐IM. These results further validate the genotyping results we obtained based on the phylogenetic structure. In addition, epitope prediction indicated that there are seven potential epitopes in PCV3a and PCV3b (Table S4, Supporting Information). For PCV3, the epitopes span almost the whole surface of the protein, which are indicated with different colors in **Figure** **5**. The detailed information about the epitopes is shown in Table S4 (Supporting Information). Combining epitope prediction with amino acid analysis, we found that amino acid site 24 of ORF2 (corresponding to site 320 of the complete coding sequence) is located in a predicted epitope region. As mentioned before, site 24 of PCV3a and PCV3b was different and, as seen in Figure 5, the epitope structures of PCV3a and PCV3b are different. This may indicate that the antigenicity of PCV3a and PCV3b is different. Further experimental studies are warranted to verify the differences in the immunogenicity of the predicted epitope regions.

### 2.5 Selection Analysis of PCV3 Complete Coding Sequences

We found that among the individual codons, 3 codons (sites 5, 122, and 320) were considered to be under positive selection with *p* values less than 0.1 (**Table** **2**). Codons 122, located in the ORF1 coding region, and 320, corresponding to the amino acid site 24 of the ORF2 coding region, were confirmed to be under positive selection by at least two methods with *p* < 0.05 by FEL (fixed effects likelihood) and MEME (mixed effects model of evolution), *p* < 0.1 by SLAC (single‐likelihood ancestor counting), and a posterior probability >0.9 by FUBAR (fast, unconstrained Bayesian approximation).

**Table 2:** Selection analysis of PCV3 complete coding sequences

Codon	FEL		SLAC		FUBAR		MEME	
	dN‐dS	*p*‐value	dN‐dS	*p*‐value	dN‐dS	Post.Pro	w^+^	*p*‐value
5	2.14	0.33	0.55	0.67	0.2194	0.6895	55.151	**0.0238**
122	14.35	**0.0048**	3.24	**0.021**	5.9994	**0.9977**	14.0822	**0.00829**
320	5.18	**0.067**	1.35	0.2	1.841	**0.955**	5.0865	0.08878

The bold representing the *p* < 0.05 and posterior probability >0.9, with significant difference.

## 3 Discussion

Since the discovery of PCV3 in 2015, most studies have focused on the genetic characterization of individual isolates and on clinical and epidemiological investigations.^22^, ^23^, ^25^, ^38^ Here, we provide new insight into the origin, epidemiology, and evolution of PCV3. We determined, with better accuracy than previous reports, the origin of PCV3 and proposed an evolutionary pathway that led to the emergence of PCV3 worldwide. First, we used conserved coding sequences (ORF1) to trace the potential origin, which was a better and more accurate approach than those previously applied to complete genomes.^21^, ^24^, ^26^ We found that clade 1 bat CVs which were isolated in China from 2011 to 2013 shared the most recent common ancestor with PCV3. Interestingly, we found several differences with previous reports. First, the pig NW2/USA/2009 strain clustered with the PCV3 clade, which was previously reported to sit as a sole outgroup in the phylogeny.^24^ Therefore, we assume that the emergence of PCV3 was before its first detection in 2009 in pigs. However, the accurate origin and divergence time need deep epidemiological surveys to be confirmed. Second, compared to previous studies,^21^, ^24^, ^26^ more reference sequences were included and we confirmed that PCV3 actually diversified from a clade 1 bat CVs.^35^ Furthermore, in accordance with a previous study, we found that PCV1 and PCV2 were closely related to the clade 2 Bat CVs,^35^ but not birds.^13^ Overall, clade 1 bat CVs are the most likely origin of PCV3 based on the currently available sequences. Generally, bats are considered to be important reservoirs of novel emerging infectious diseases. Circovirus host jumps may be a risk for both the pig industry and public health, similar to the recently reported bat‐origin coronavirus detected in pigs.^39^, ^40^

Given that previously recognized NJ *p*‐distance methods that identified PCV2 genotype were questioned by Franzo et al.,^15^ we explored the phylogenetic history of PCV3 based on recent outbreak strains.^41^, ^42^ For the genotype identification of PCV3, three different algorithms, NJ, ML, and MCC, were used and two stable clades were identified (PCV3a and PCV3b). Additionally, within the PCV3a clade, two stable subclades and flexible IM clades were identified. Compared to previous studies,^24^, ^31^ the topologies of the trees provided here, deduced using complete coding sequences, were more stable and accurate (Figure S4, Supporting Information). On the other hand, amino acid analysis revealed that S122A and A320V were vital in differentiating the PCV3a and PCV3b clades. Although genotype identification combined with amino acid analysis has been reported by Fu et al.^24^ and Fux et al.,^31^ it is essential to note that: i) both studies included the low sequencing quality strain KY418606 (which has been revised after the two studies were published), misleading the topology of the phylogeny; ii) the subgenotype identification of the two studies was not based on full genomes, while complete coding sequences were used here; and iii) the stable topologies observed in our phylogenetic trees reconstructed with complete coding sequences were confirmed by principle component analysis (PCA) using different sequences in one of our unpublished studies. Therefore, accurate phylogenetic trees and genotype identification of PCV3 should be deduced using unrooted ML trees of complete coding sequences in the future.

Phylodynamic analysis revealed that the divergence of PCV3a was earlier than PCV3b. We believe that the short divergence time identified for PCV3a and PCV3b is due to a lack of adequate number of PCV3 sequences. The skyline plot revealed a rapid increase and expansion of the PCV3 population from late 2013 to early 2014. In addition, during this period, PCV3a and PCV3b were formed. The initial phase of an epidemic is usually associated with exponential growth (corresponding to Re = R0). Re must always be less than or equal to R0 by definition. If the value of R0 or Re is more than one, an epidemic may occur.^43^ We estimated that the Re of PCV3a and 3b were 3.18 (CI: 0.82–7.96) and 1.82 (CI: 0.63–4.86) in 2016, respectively. Additionally, the high substitution rate of PCV2 within single stranded DNA virus, enabled it to experience an evolutionary dynamics similar to single stranded RNA viruses and, therefore, enhanced the global emergence of PCVD.^13^ Interestingly, the substitution rate of PCV3 was relatively higher than PCV2, which might facilitate PCV3 to adapt to different biological conditions and pose a greater threat to the swine population. Overall, these estimated results were consistent with the actual epidemic of PCV3 and indicate that both PCV3a and 3b have high potential for widespread transmission in the future. Thus, epidemiological investigation to prevent this novel disease is crucial. Moreover, our analysis revealed epidemiological features associated with the geographical distribution in China, which might indicate that once PCV3 strains establish into specific areas, they might adapt to the local hosts. Importantly, amino acid site 24 of the ORF2 protein (codon of 320 in the complete coding region) was predicted as a potential epitope and to be under positive selection. This site may be pivotal to the escape of PCV3 from the host immune system leading to a prolonged period of circulation and the divergence of the PCV3a to the PCV3b clade.^44^

In conclusion, we confirmed the more accurate bat origin of PCV3, and provided a comprehensive genotype identification. Additionally, we report for the first time that PCV3 replaced PCV2 and became the single stranded DNA virus with highest substitution rate. The rapid increase population and relative high Re value reveals the possibility of a continuous outbreak in the future. Overall, our study aids the understanding of ongoing PCV3 cases worldwide and will guide future efforts to develop effective preventive and control measures.

## 4 Experimental Section

*Sequence Datasets* **—** *Multiple Circoviruses Datasets*: All the sequences were obtained from the GenBank database of NCBI (November 2017, Table S1, Supporting Information) (https://www.ncbi.nlm.nih.gov). A total of 57 PCV3 strains were analyzed in this study. In addition, 101 ORF1 coding sequences of different circoviruses which were previously reported,^12^, ^21^, ^24^, ^35^ including 13 PCV2 strains, 2 PCV1 strains, 9 bat CVs, 20 other host circoviruses, and 1 strain detected from a pork sample were also included.

*Sequence Datasets* **—** *PCV3 Datasets*: A total of 57 complete coding sequences (ORF1 + ORF2) were analyzed. The noncoding regions were deleted and the coding sequences were split into two representing ORF1 (Rep) and ORF2 (Cap). Given that ORF2 is in the opposite orientation, the individual ORFs were downloaded and then concatenated. Additionally, 109 individual ORF2 gene encoding capsid proteins were included.

*Recombination Detection*: Recombination analysis was performed based on the approaches applied in RDP4.^45^ A total of seven methods were applied, including GENECONV,^46^ RDP,^47^ Chimaera,^48^ SiScan,^49^ 3Seq,^50^ MaxChi,^51^ LARD.^52^ Recombination had to be confirmed by at least four of the seven methods with *p* value cut‐off of 0.05. Bonferroni correction was applied throughout the analysis.

*Sequence Alignment and Model Test*: Sequences were aligned by ClustalW implemented in MEGA 7.^53^ The best substitution model was selected by jModelTest according to the Bayesian information criterion (BIC) score.

*Origin and Phylogenetic Analysis*: To date the origin of PCV3, a ML tree was reconstructed with 101 amino acid sequences of the Rep protein using RAxML with PROTCATLG model and 1000 bootstrap replicates. The lineages closely related to PCV3, PCV2, and PCV1 were used to infer the divergence time. The MCC trees were inferred by BEAST (v1.8.4) with the nucleotide sequences of the ORF1 gene using the Hasegawa–Kishino–Yano model with four discrete gamma categories (HKY + G). Additionally, to identified PCV3 genotypes, three distinct methods were used including: MCC tree using BEAST (v1.8.4),^54^ ML tree using RAxML,^55^ and NJ tree using MEGA7.^53^ The *p*‐distance methods were used to infer the NJ tree with 1000 bootstraps replication.

*Evolutionary Dynamics of PCV3*: Bayesian MCMC methods within the BEAST (v1.8.4) package were used to estimate the time of the most recent common ancestor (tMRCA) and the evolutionary rates.^56^ The nucleotide substitution model was general time reversible substitution model with a proportion of invariant sites and gamma distributed rate heterogeneity (GTR+I+G), assuming an uncorrected relaxed clock (lognormal). A Bayesian skyline coalescent model was set to estimate the efficient population size. Two independent runs were operated with a chain length of 1 × 10^8^ generations and sampled at every 10 000 generations.^57^ Convergence was estimated based on the software Tracer (v1.6) (http://tree.bio.ed.ac.uk/software/tracer/) after a burn‐in of 10%. Parameters with effective sampling size (ESS) > 200 were accepted. The final MCC trees were replayed in Figtree (v1.4.3) (http://tree.bio.ed.ac.uk/software/figtree/). BaTS (Bayesian tip‐significance testing) was used to analyze the correlation among the PCV3 sequences and geographical structure.^58^ The countries of each sequence were used as the taxon labels, such as China, USA, Korea, Italy, and Brazil. The AI and PS statistic were calculated using the BEAST MCC tree to obtain statistical support. A *p* value less than 0.05 was considered statistically significant. Also, the worldwide geographical distribution of PCV3 was marked in the map (Figure S3, Supporting Information).

*Effective Reproduction Number (Re)*: Re values were calculated using follow formula (1)

(1) $$R=\frac{\lambda }{\left(\mu +\rho \right)}$$

Among these parameters, *R* is the effective reproduction number, λ is the birth rate, μ is the death rate, ρ is the sampling probability.^59^ BEAST (v2.4.7) was used to estimate the Re. The GTR+G nucleotide substitution model was chosen and the Birth death skyline contemporary model with sampling proportion was set to 0. The chain length was 1 × 10^7^, every 1000 generations resampled one time. Two independent runs were performed and log files were combined using LogCombiner.

*Predicting the Structure of PCV3a and PCV3b*: The tertiary structure models of PCV3a and PCV3b ORF2 protein were built using the I‐TASSER online tool^60^ (https://zhanglab.ccmb.med.umich.edu) based on the structure of PCV2. PCV3a (Strain: KX778720 PCV3‐US/MO2015) and PCV3b (Strain:MF589105 PCV3/CN/Guangdong‐SG1/2016) sequences were used for the comparative analysis of epitopes and viral structure. The viral surface and structure were depicted in PyMOL (v1.5.0.4).^61^ The epitopes of the PCV3 cap protein were predicted using the online server: (http://www.cbs.dtu.dk/services/BepiPred/) (epitope threshold set up at 0.5).

*Selection Analysis*: The detection of selection on the complete coding sequences of PCV3 was performed using DATAMONKEY (http://www.datamonkey.org/). The methods used to investigate positive codon sites included FEL, SLAC, FUBAR, MEME,^62^, ^63^, ^64^ the branch site REL and the GA‐branch site models were chosen to determine the selection pressure on the individual branches.^65^, ^66^ Methods with *p* < 0.1 in SLAC, *p* < 0.05 in FEL and MEME and the posterior probability >0.9 in FUBAR, were considered to be more conservative positive selection pressure.

## Conflict of Interest

The authors declare no conflict of interest.

## Supporting information

Supplementary
