# Supplementary material for: Origin, Genetic Diversity, and Evolutionary Dynamics of Novel Porcine Circovirus 3
Source: Adv Sci (Weinh). 2018 Jul 4;5(9):1800275. doi: 10.1002/advs.201800275 (PMC6145280; doi:10.1002/advs.201800275)
Supplement: Supplementary file 1 — Supplementary [file ADVS-5-1800275-s001.pdf]

Copyright WILEY-VCH Verlag GmbH & Co. KGaA, 69469 Weinheim, Germany, 2018.

ADVANCED SCIENCE
Open Access

Supporting Information

for *Adv. Sci.,* DOI: 10.1002/advs.201800275

Origin, Genetic Diversity, and Evolutionary Dynamics of Novel Porcine Circovirus 3

*Gairu Li, Wanting He, Henan Zhu, Yuhai Bi, Ruyi Wang, Gang Xing, Cheng Zhang, Jiyong Zhou, Kwok-Yung Yuen, George F. Gao, and Shuo Su**

Supporting Information

Origin, Genetic Diversity, and Evolutionary Dynamics of Novel Porcine Circovirus 3

G. Li, W. He, H. Zhu, Y. Bi, R. Wang, G. Xing, C. Zhang, J. Zhou, K.-Y. Yuen, G. F. Gao, S. Su*.

Supporting figures:

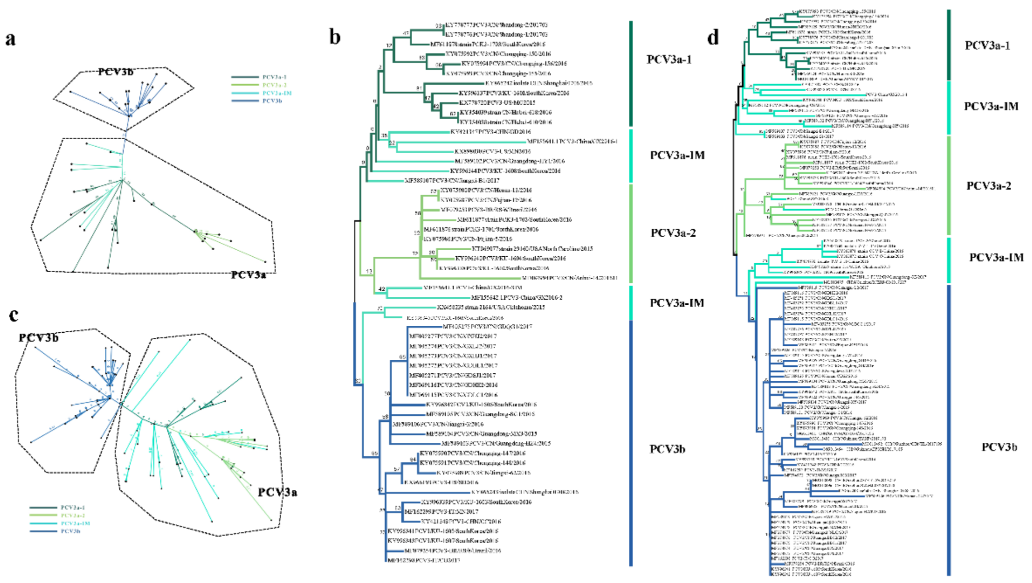

Figure S1: Phylogenetic trees of 56 ORF2 and 109 ORF2 gene of PCV3. a) ML tree of 56 ORF2 complete coding region sequences inferred by RAxML. b) the NJ tree of 56 ORF2 complete coding region, inferred by MEGA 7.0. c) the ML tree of 109 ORF2 complete coding region, inferred by RAxML. d) the NJ tree of 109 ORF2 complete coding region, inferred by MEGA 7.0.

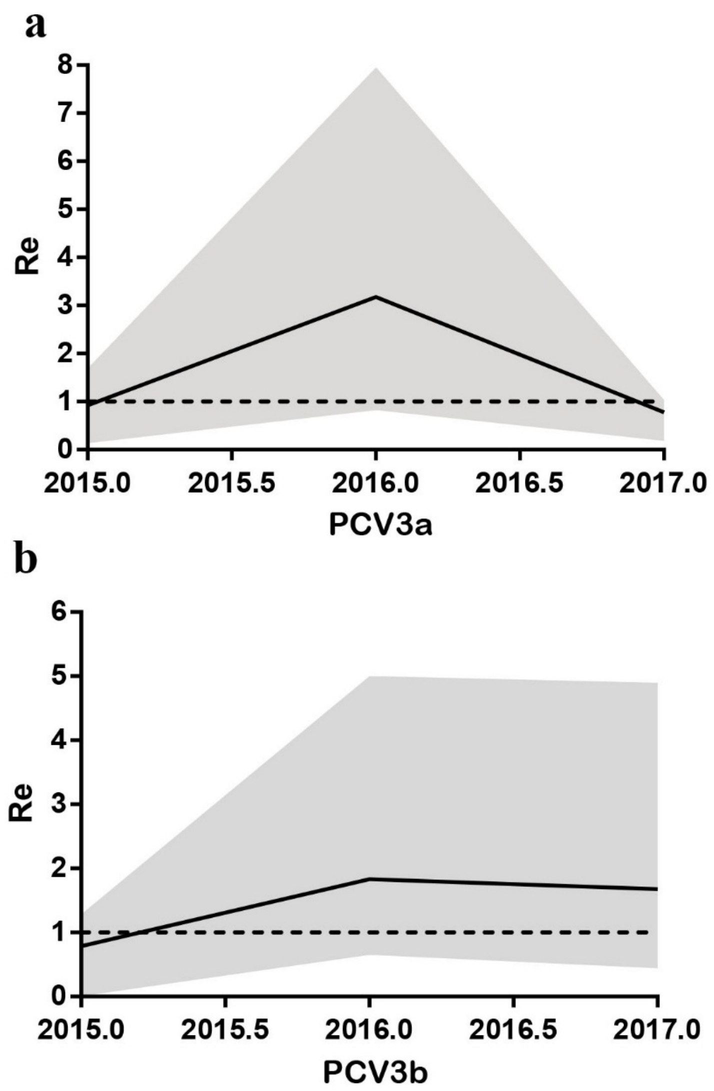

Figure S2: The Re of PCV3 in related sequences collected in 2015, 2016, and 2017. a) PCV3a, b) PCV3b.

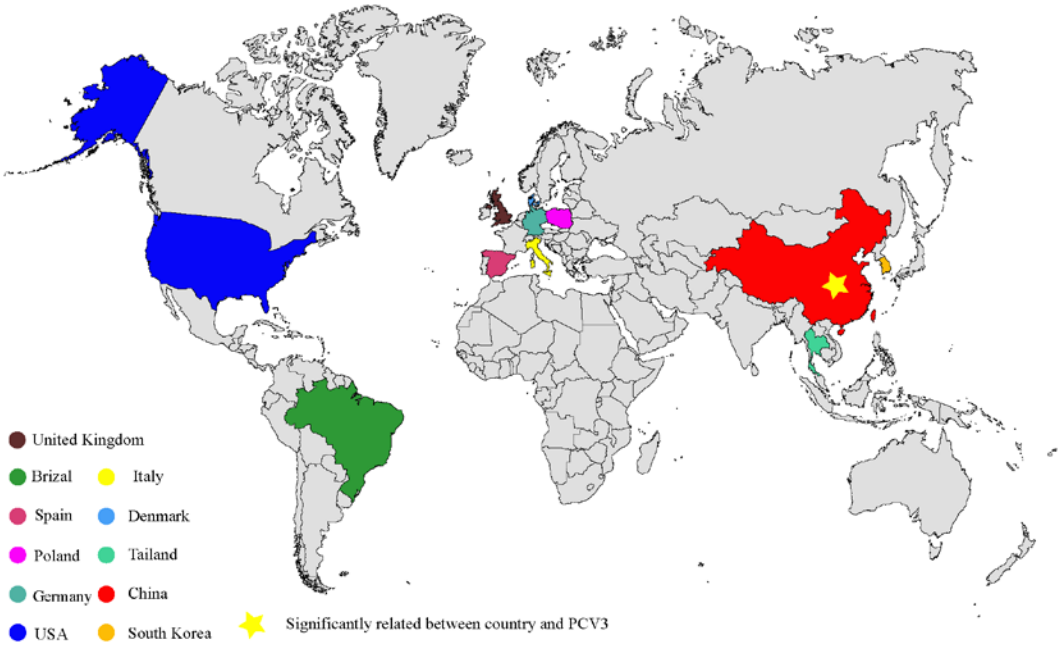

Figure S3: PCV3 distribution countries is marked in different colors and the association of geographical distribution of PCV3 in China is marked with a star.

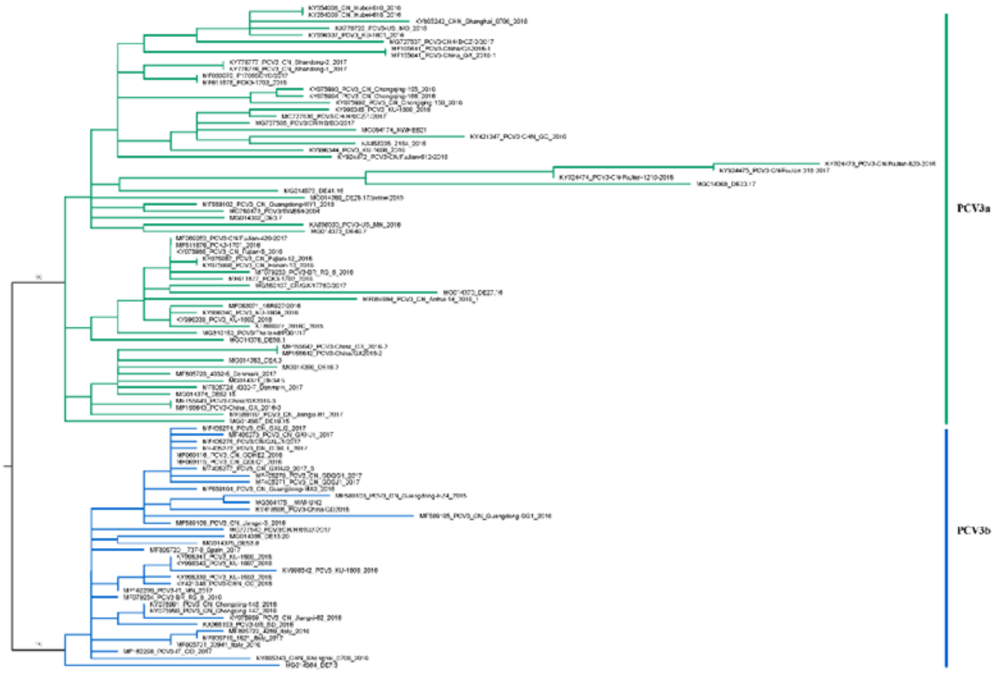

Figure S4: ML tree of 98 PCV3 complete coding sequences collected until May 6th 2018 reconstructed using RAxML. We show that if the sequence number increases, the structure of the tree is stable to form PCV3a and PCV3b with a high confidence (bootstrap = 90).

Supporting tables:

Table S1. Detailed information of the 56 complete coding sequences of PCV3.

Accession number	Strain name	Country	Year
KT869077	29160	USA	2015
KX458235	2164	USA	2015
KX778720	PCV3-US/MO	USA	2015
KX898030	PCV3-US/MN	USA	2016
KX966193	PCV3-US/SD	USA	2016
KY075986	PCV3/CN/Fujian-5	China (Fujian)	2016
KY075987	PCV3/CN/Fujian-12	China (Fujian)	2016
KY075988	PCV3/CN/Henan-13	China (Henan)	2016
KY075989	PCV3/CN/Jiangxi-62	China (Jiangxi)	2016
KY075990	PCV3/CN/Chongqing-147	China (Chongqing)	2016
KY075991	PCV3/CN/Chongqing-148	China (Chongqing)	2016
KY075992	PCV3/CN/Chongqing-150	China (Chongqing)	2016
KY075993	PCV3/CN/Chongqing-155	China (Chongqing)	2016
KY075994	PCV3/CN/Chongqing-156	China (Chongqing)	2016
KY354038	CN/Hubei-610	China (Hubei)	2016
KY354039	CN/Hubei-618	China (Hubei)	2016
KY421347	PCV3-CHN/GD	China (Guangdong)	2016
KY421348	PCV3-CHN/CC	China	2016
KY778776	PCV3/CN/Shandong-1	China (Shangdong)	2017
KY778777	PCV3/CN/Shandong-2	China (Shangdong)	2017
KY865242	CHN Shanghai 0706	China (Shanghai)	2016
KY865243	CHN Shanghai 0708	China (Shanghai)	2016
KY996337	PCV3/KU-1601	South Korea	2016
KY996339	PCV3/KU-1603	South Korea	2016
KY996340	PCV3/KU-1604	South Korea	2016
KY996341	PCV3/KU-1605	South Korea	2016
KY996342	PCV3/KU-1606	South Korea	2016
KY996343	PCV3/KU-1607	South Korea	2016
KY996344	PCV3/KU-1608	South Korea	2016
KY996345	PCV3/KU-1609	South Korea	2016
KY996388	PCV3/KU-1602	SouthKorea	2016
MF069115	PCV3/CN/GDLC1	China (Guangdong)	2016
MF069116	PCV3/CN/GDHE2	China (Guangdong)	2016
MF079253	PCV3-BR/RS/6	Brazil	2016
MF079254	PCV3-BR/RS/8	Brazil	2016
MF084994	PCV3/CN/Anhui-14	China (Anhui)	2016
MF155641	PCV3-China/GX2016-1	China (Guangxi)	2016

MF155642	PCV3-China/GX2016-2	China (Guangxi)	2016
MF155643	PCV3-China/GX2016-3	China (Guangxi)	2016
MF162298	PCV3-IT/CO/2017	Itaily	2017
MF162299	PCV3-IT/MN	Itaily	2017
MF405271	PCV3/CN/GDSJ1	China (Guangdong)	2017
MF405272	PCV3/CN/GDBL1	China (Guangdong)	2017
MF405273	PCV3/CN/GXHJ1	China (Guangxi)	2017
MF405274	PCV3/CN/GXLJ2	China (Guangxi)	2017
MF405275	PCV3/CN/GDQG1	China (Guangxi)	2017
MF405277	PCV3/CN/GXHJ2	China (Guangxi)	2017
MF589102	PCV3/CN/Guangdong-HY1	China (Guangdong)	2016
MF589103	PCV3/CN/Guangdong-HZ4	China (Guangdong)	2015
MF589104	PCV3/CN/Guangdong-MX3	China (Guangdong)	2015
MF589105	PCV3/CN/Guangdong-SG1	China (Guangdong)	2016
MF589106	PCV3/CN/Jiangxi-3	China (Jiangxi)	2016
MF589107	PCV3/CN/Jiangxi-B1	China (Jiangxi)	2017
MF611876	PCK3-1701	South Korea	2016
MF611877	PCK3-1702	South Korea	2016
MF611878	PCK3-1703	South Korea	2016

The information of other strains chosen in this study.

**Accession number**	**Strain name**	**Country**	**Year**
AF055392	Porcine circovirus Type II	Canada	-
AF055394	Porcine circovirus Type II	France	-
AF071878	Beak and feather disease virus	-	-
AF080560	Beak and feather disease virus	-	-
AF252610	Columbid circovirus	-	-
AJ301633	Canary circovirus	-	-
AJ304456	Goose circovirus	Germany	-
AY228555	DuCV	Brandenburg	2002
DQ100076	33753-52	USA	-
DQ146997	4-1131	Australia	-
DQ172906	Starling circovirus	Spain	2005
DQ845075	Finch circovirus	-	-
EU056309	H51	Germany	2006
EU056310	Sw4	Germany	2006
EU148503	DK1980PMWSfree	Denmark	-
EU148504	DK1987PMWSfree	Denmark	-
FJ667585	PCV2-RP3	China	2007
GQ404851	Chimp17	Rwanda	2002
GU247991	FJGT0602	China	2006
GU799606	Barbel circovirus	Hungary	2008

HQ831539	PT-20683-09	Portugal: Montijo	2009
JQ814849	Rhinolophus ferrumequinum circovirus 1	China	2011
JQ821392	214	USA	2011
JX512854	S00-1966/4-3	Switzerland	2000
JX512855	S98-305/2	Switzerland	1998
JX534236	HALY12	China	2012
JX982219	KSRY1103	China: Kunshan	2011
KC241982	UCD1-1698	USA	2011
KC241983	UCD3-478	USA	2011
KC339249	XOR7	Myanmar	2008
KC990120	90507	China	2009
KJ408798	PCV1-Eng-1970	United Kingdom	1990
KJ641711	BtMr-CV/GD2012	China	2012
KJ641716	BtPspp.-CV/GD2012	China	2012
KJ641723	BtRs-CV/HuB2013	China	2013
KJ641724	BtRa-CV/JS2013	China	2013
KJ641727	BtPa-CV-1/NX2013	China	2013
KJ641741	BtRf-CV-61/YN2010	China	2010
KJ641742	BtRf-CV-62/YN2010	China	2010
KP229370	CD13088	Taiwan: Chiayi	2013
KP793918	8454V25-1	Germany	2010
KT795287	MEX/41238/2014	Mexico: Veracruz	2014
KT795290	USA/45358/2015	USA	2015
KY363870	CCV-A	China	2016
KY363871	CCV-B	China	2016
KY363872	CCV-C	China	2016
KY484769	YiY-1-11	China	2016
KY484770	YiY-2-11	China	2016
MF374971	PCV3/CN/Guangxi002/2017	China	2017
MF374972	PCV3/CN/Guangxi003/2017	China	2017
MF374973	PCV3/CN/Guangxi004/2017	China	2017
MF374974	PCV3/CN/Guangxi005/2017	China	2017
MF374975	PCV3/CN/GuangxiBB01/2017	China	2017
MF374976	PCV3/CN/GuangxiBB02/2017	China	2017
MF374977	PCV3/CN/GuangxiNNLQ/2017	China	2017
MF374978	PCV3/CN/GuangxiNNWM/2017	China	2017
MF374979	PCV3/CN/GuangxiQZSS/2017	China	2017
MF383379	PCV3/CN/Guangxi001/2017	China	2017
MF496982	PCV3/CN/Hainan001/2017	China	2017
MF589102	PCV3/CN/Guangdong-HY1/2016	China	2016
MF589108	PCV3/CN/Fujian-FZ/2015	China	2015
MF589109	PCV3/CN/Fujian-HWK1/2016	China	2016

MF589110	PCV3/CN/Fujian-HWK2/2016	China	2016
MF589111	PCV3/CN/Fujian-KP1/2016	China	2016
MF589112	PCV3/CN/Guangdong-CH/2016	China	2016
MF589113	PCV3/CN/Guangdong-GZ/2017	China	2017
MF589114	PCV3/CN/Guangdong-HY/2016	China	2016
MF589115	PCV3/CN/Guangdong-JM1/2016	China	2016
MF589116	PCV3/CN/Guangdong-MX1/2016	China	2016
MF589117	PCV3/CN/Guangdong-SG/2016	China	2016
MF589118	PCV3/CN/Guangdong-X1/2016	China	2016
MF589119	PCV3/CN/Guangxi-L2/2017	China	2017
MF589120	PCV3/CN/Guangxi-LD4/2016	China	2016
MF589121	PCV3/CN/Guangxi-LD5/2016	China	2016
MF589122	PCV3/CN/Guangxi-NK/2015	China	2015
MF589123	PCV3/CN/Guangxi-WZ/2016	China	2016
MF589124	PCV3/CN/Hunan-B8/2016	China	2016
MF589125	PCV3/CN/Hunan-CD33/2016	China	2016
MF589126	PCV3/CN/Hunan-CZ/2017	China	2017
MF589127	PCV3/CN/Hunan-HWF2/2017	China	2017
MF589128	PCV3/CN/Hunan-HWF3/2017	China	2017
MF589129	PCV3/CN/Hunan-XHD2/2016	China	2016
MF589130	PCV3/CN/Jiangxi-1/2016	China	2016
MF589131	PCV3/CN/Jiangxi-G1/2016	China	2016
MF589132	PCV3/CN/Jiangxi-QN3/2016	China	2016
MF589133	PCV3/CN/Jiangxi-S1/2017	China	2017
MF589134	PCV3/CN/Jiangxi-XY/2017	China	2017
MG008470	CHN/Guizhou/BJZHJ-01/2017225	China	2017
MG013489	CHN/Guizhou/GYKY-01/2017215	China	2017
MG013490	CHN/Guizhou/GYKY-02/2017219	China	2017
MG013491	CHN/Guizhou/QNDSH/2017156	China	2016
MG013492	CHN/Guizhou/QDNKL/2017199	China	2017
MG013493	CHN/Guizhou/ZYXP/2017193	China	2016
MG013494	CHN/Guizhou/ZYXSH/2017185	China	2016
MG013495	CHN/Guizhou/BJZHJ-02/2017227	China	2017
MG013496	CHN/Guizhou/BJZHJ-03/2017250	China	2017

Note: “-” means that this information was not available on NCBI.

Table S2. The mean and 95% HPD nucleotide substitution rate and tMRCA of PCV1, PCV2, PCV3, ORF1 and ORF2 of PCV3 each clade.

**Name**	**Substitution rate**		**tMRCA**	
	**mean**	**95%HPD**	**mean**	**95%HPD**
**PCV1**	1.15×10^{-5}		-	-
**PCV2**	1.2×10^{-3}	8.23×10^{-4}-1.61×10^{-3}	-	-
**PCV3**	1.69×10^{-3}	9.99×10^{-4}-2.45×10^{-3}		
**PCV3a-1 ORF1**	1.1×10^{-3}	1.0×10^{-4}-2.5×10^{-3}	2013.61	2012.09-2014.77
**PCV3a-2 ORF1**	1.1×10^{-3}	1.0×10^{-4}-2.5×10^{-3}	2013.48	2011.97-2014.63
**PCV3b ORF1**	1.4×10^{-3}	1.0×10^{-4}-3.5×10^{-3}	2013.25	2011.51-2014.52
**PCV3a-1 ORF2**	3.3×10^{-3}	5.0×10^{-4}-4.6×10^{-3}	2014.00	2013.19-2014.72
**PCV3a-2 ORF2**	2.5×10^{-3}	4.0×10^{-4}-4.8×10^{-3}	2013.91	2013.05-2014.64
**PCV3b ORF2**	3.2×10^{-3}	6.0×10^{-4}-7.2×10^{-3}	2013.79	2012.85-2014.55

Table S3. Amino acid analysis for PCV3 complete coding sequences.

Genotype/ Position	PCV3a-1 (11)	PCV3a-2 (10)	PCV3b (25)	PCV3a-IM (10)
122	S(4)	S(0)	**A(0)**	S(1)
320	A(1)	A(0)	**V(0)**	A(2)
323	R(0)	R(0)	**K(0)**	K(5)
373	S(2)	**T(0)**	S(1)	S(0)
446	I(2)	**L(0)**	I(0)	I(0)

Note: from amino site 297 is the cap coding region.

Table S4. Predicted epitopes of PCV3a and PCV3b.

Cap protein	Epitope	Residue(aa)	Epitope sequence
**PCV3a**	**A**	5~25	AIFRRRPRPRRRRRHRRRYAR
	**B**	42~61	KYSTMNVISVGTPQNNKPWH
	**C**	71~81	EWETAISFEYY
	**D**	95~105	SPAQQTKTMFG
	**E**	114-159	AWTTNTWLQDDPYAESSTRKVMTSKKK HSRYFTPKPILAGTTSAHP
	**F**	169-175	PTPWLNT
	**G**	191-204	VPEKTGMTDFYGTK
**PCV3b**	**A**	5~26	AIFRRRPRPRRRRRHRRRYVRR
	**B**	42~60	KYSTMNVISVGTPQNNKPW
	**C**	71~81	EWETAISFEYY
	**D**	95~105	SPAQQTKTMFG
	**E**	114-159	AWTTNTWLQDDPYAESSTRKVMTSKKK HSRYFTPKPILAGTT
	**F**	169-174	PTPWLN
	**G**	191-204	VPEKTGMTDFYGTK
